# Supplementary material for: “Smart” Triiodide Compounds: Does Halogen Bonding Influence Antimicrobial Activities?
Source: Pathogens. 2019 Oct 10;8(4):182. doi: 10.3390/pathogens8040182 (PMC6963602; doi:10.3390/pathogens8040182)
Supplement: Supplementary file 1 [file pathogens-08-00182-s001.pdf]

pathogens

MDPI

Supplementary Information

"Smart" Triiodide Compounds: Halogen Bonding Has Influence on Antimicrobial Activities ?

Zehra Edis [1,*], Samir Haj Bloukh [1], Hamed Abu Sara [1], Hanusha Bhakhoa [2], Lydia Rhyman [2,3], and Ponnadurai Ramasami [2,3,*]

Zehra Edis [1,*], Samir Haj Bloukh [1], Hamed Abu Sara [1], Hanusha Bhakhoa [2], Lydia Rhyman [2,3] and Ponnadurai Ramasami [2,3,*]

[1] College of Pharmacy and Health Science, Ajman University, Ajman PO Box 346, United Arab Emirates; s.bloukh@ajman.ac.ae (S.H.B.); h.abusara@ajman.ac.ae (H.A.S.)
[2] Computational Chemistry Group, Department of Chemistry, Faculty of Science, University of Mauritius, Réduit 80837, Mauritius; hbhakhoa@gmail.com (H.B.); lyd.rhyman@gmail.com (L.R.);
[3] Department of Chemical Sciences, University of Johannesburg, Doornfontein, Johannesburg 2028, South Africa
* Correspondence: z.edis@ajman.ac.ae; (Z.E.); p.ramasami@uom.ac.mu; (P.R.)

Computational details

Geometry optimisation and single-point computations were conducted in solvents (water and n-octanol) using the B3LYP-D3 functional [S1-S3] in conjunction with the 6-311G(d,p) basis set [S4, S5] in view to calculate the lipophilicity of relevant species studied to this work.

Geometry optimisation was followed by analytic Hessian computation using the same methods. The absence of negative Hessian eigenvalues confirmed the stationary points as minima on the potential energy hypersurfaces.

Solvent effect was taken into account based on the polarisable continuum model [S6]. The classical descriptor for lipophilicity is the log $P_{o/w}$ (partition coefficient between n-octanol and water) [S7]. The log $P_{o/w}$ values were obtained using equation (1):

$$\log P_{o/w} = \frac{-\Delta G}{2.303RT} \quad (1)$$

where R is the universal gas constant (8.314 J. $K^{-1}.mol^{-1}$), T is the system temperature (298.15 K) and ΔG (in $J.mol^{-1}$) is the difference between the solvation absolute Gibbs free energies in water (G_{water}) and in n-octanol ($G_{n\text{-}octanol}$). The log $P_{o/w}$ values for the species were also calculated via the SwissADME [S7] and the ALOGPS 2.1 program [S8].

All computations were performed by means of resources provided (Gaussian 09 package [S9]) by SEAGrid [S10-S14].

Lipophilicity

Table S1. log $P_{o/w}$ of species relevant to this study obtained using SwissADME web tool [S7].

Species	MW[a]	iLOGP	XLOGP3	WLOGP	MLOGP	Silicos-IT log *P*	Consensus log *P*
12-crown-4	176.21	2.14	–0.04	0.07	–0.83	1.59	0.59
$[Na(12\text{-crown-}4)_2]^+$	375.41	–3.76	–1.55	0.13	–1.83	–6.98	–2.8
$[Na(12\text{-crown-}4)_2]I_3$	756.12	–1.85	1.03	–1.09	–0.44	–6.98	–1.87
$[Na(12\text{-crown-}4)_2][(I_3)_2]^-$	1136.84	–1.61	3.61	–2.32	0.79	–6.98	–1.3
Chitosan	1526.45	1.88	–21.4	–20.53	–17.69	–21.13	–15.77

a = molecular weight

Cartesian coordinates

$[Na(12\text{-crown-}4)_2][(I_3)_2]^-$ -- in octanol

63
symmetry c1

I	-6.144174000	-0.281953000	-0.377278000
I	-3.244821000	0.418988000	-0.811012000
I	-9.076761000	-0.986584000	0.080928000
I	3.862480000	-2.750074000	-0.022517000
I	1.207755000	-3.759342000	0.958782000
I	6.496721000	-1.603871000	-1.019216000
Na	1.800014000	2.347772000	0.319865000
O	1.742460000	4.154206000	2.024952000
C	2.998783000	4.175591000	2.705660000
H	2.976056000	4.875574000	3.549746000
H	3.718282000	4.532852000	1.966868000
C	3.420133000	2.794370000	3.186122000
H	4.386929000	2.865943000	3.702607000
H	2.690372000	2.393614000	3.898917000
O	3.528626000	1.956688000	2.039262000
C	3.570718000	0.548064000	2.291843000
H	4.251812000	0.318650000	3.120129000
H	3.962137000	0.107365000	1.375351000
C	2.193327000	-0.033487000	2.572693000
H	2.278883000	-1.117334000	2.719773000
H	1.760484000	0.400907000	3.482411000
O	1.375614000	0.244124000	1.440433000
C	-0.030694000	0.071647000	1.621767000
H	-0.244588000	-0.823201000	2.215873000
H	-0.437397000	-0.069504000	0.620778000
C	-0.674573000	1.293886000	2.257492000
H	-1.758007000	1.137301000	2.332470000
H	-0.280370000	1.459932000	3.266929000
O	-0.396879000	2.413747000	1.414797000
C	-0.594905000	3.698916000	1.999978000
H	-1.517835000	3.726916000	2.591766000
H	-0.693892000	4.387340000	1.158750000
C	0.585905000	4.123406000	2.861416000
H	0.395634000	5.118415000	3.285365000
H	0.724328000	3.424611000	3.693823000
O	0.588600000	3.868533000	-1.222189000
C	1.428386000	4.669985000	-2.048412000
H	0.886962000	5.559499000	-2.397728000
H	1.758186000	4.111016000	-2.931318000
C	2.624262000	5.098474000	-1.210630000
H	3.257341000	5.784778000	-1.786481000
H	2.269830000	5.610547000	-0.314412000

O	3.378703000	3.984528000	-0.732648000
C	4.269281000	3.387995000	-1.677176000
H	5.175227000	3.999387000	-1.784731000
H	3.797972000	3.311198000	-2.663606000
C	4.639736000	2.008105000	-1.155305000
H	5.374167000	1.533960000	-1.814930000
H	5.074854000	2.100771000	-0.159044000
O	3.492954000	1.176828000	-0.988369000
C	3.009956000	0.535166000	-2.167895000
H	3.665902000	-0.300988000	-2.439647000
H	2.985482000	1.237851000	-3.009422000
C	1.613874000	0.013848000	-1.863259000
H	1.218836000	-0.533532000	-2.727205000
H	1.661003000	-0.659460000	-1.008023000
O	0.724992000	1.062652000	-1.469912000
C	0.156463000	1.833336000	-2.523686000
H	-0.654964000	1.276420000	-3.010331000
H	0.907627000	2.071552000	-3.285811000
C	-0.409247000	3.103029000	-1.904286000
H	-0.909279000	3.705269000	-2.672472000
H	-1.134660000	2.832672000	-1.136932000

$[Na(12\text{-crown-}4)_2][(I_3)_2]^-$ -- in water

63
symmetry c1

I	-3.968887000	-0.433067000	0.308919000
I	-3.898293000	-1.825848000	3.007883000
I	-3.998802000	0.925719000	-2.378710000
I	2.110205000	-2.889989000	-0.788081000
I	-0.618808000	-2.612477000	-2.002946000
I	4.881682000	-3.050363000	0.477937000
Na	1.388834000	2.506822000	0.323873000
O	1.301196000	3.939594000	2.381677000
C	2.604190000	3.983393000	2.966446000
H	2.583011000	4.498475000	3.934522000
H	3.211360000	4.560342000	2.267992000
C	3.207039000	2.596990000	3.132313000
H	4.197094000	2.679778000	3.600203000
H	2.578487000	1.982324000	3.785947000
O	3.318502000	2.013174000	1.835461000
C	3.541493000	0.600317000	1.809618000
H	4.313570000	0.310782000	2.532621000
H	3.905049000	0.384015000	0.804648000
C	2.270037000	-0.191579000	2.069402000
H	2.490393000	-1.263683000	2.000466000

H	1.885493000	0.011719000	3.075375000
O	1.314977000	0.179515000	1.078129000
C	-0.034657000	-0.192381000	1.366019000
H	-0.079606000	-1.192595000	1.812251000
H	-0.549268000	-0.224489000	0.405803000
C	-0.705585000	0.817936000	2.279824000
H	-1.736339000	0.509307000	2.483706000
H	-0.175325000	0.876818000	3.236623000
O	-0.685639000	2.079579000	1.608683000
C	-0.965419000	3.215362000	2.427696000
H	-1.831429000	3.028847000	3.074284000
H	-1.207320000	4.018240000	1.729856000
C	0.231503000	3.626906000	3.272535000
H	-0.030820000	4.504505000	3.878142000
H	0.516399000	2.821147000	3.957645000
O	-0.114602000	4.197198000	-0.708418000
C	0.556792000	5.318835000	-1.281652000
H	-0.118154000	6.183967000	-1.328417000
H	0.885376000	5.097080000	-2.303155000
C	1.748386000	5.652353000	-0.397354000
H	2.251549000	6.550092000	-0.775329000
H	1.403384000	5.838906000	0.620708000
O	2.663083000	4.560280000	-0.286716000
C	3.573036000	4.398928000	-1.373103000
H	4.369635000	5.152963000	-1.320456000
H	3.061665000	4.520198000	-2.334227000
C	4.181382000	3.009141000	-1.257660000
H	4.942071000	2.868841000	-2.035001000
H	4.647220000	2.901557000	-0.277295000
O	3.193434000	1.978546000	-1.316340000
C	2.717589000	1.652784000	-2.622698000
H	3.452346000	1.032367000	-3.152689000
H	2.554436000	2.558760000	-3.216705000
C	1.421788000	0.874979000	-2.456869000
H	1.049324000	0.550215000	-3.435363000
H	1.605204000	-0.008679000	-1.846684000
O	0.428035000	1.625038000	-1.755137000
C	-0.290449000	2.576992000	-2.539333000
H	-1.025828000	2.067766000	-3.173170000
H	0.389617000	3.139310000	-3.188706000
C	-1.014865000	3.509189000	-1.581936000
H	-1.631653000	4.217975000	-2.147357000
H	-1.665621000	2.923156000	-0.932133000

References

S1. Becke, A.D. Density-functional thermochemistry. III. The role of exact exchange. *J. Chem. Phys.* **1993**, *98*, 5648–5652.

S2. Lee, C.; Yang, W.; Parr, R.G. Development of the Colle-Salvetti correlation-energy formula into a functional of the electron density. *Phys. Rev. B* **1988**, *37*, 785–789.

S3. Grimme, S.; Antony, J.; Ehrlich, S.; Krieg, H. A consistent and accurate ab initio parametrization of density functional dispersion correction (DFT-D) for the 94 elements H-Pu. *J. Chem. Phys.* **2010**, *132*, 154104.

S4. Hehre, W.J.; Radom, L.; Schleyer, P.v.R.; Pople, J. Ab initio Molecular Orbital Theory, Wiley: New York, 1986.

S5. Adamo, C.; Barone, V. *J. Chem. Phys.* **1999**, *110*, 6158–6170.

S6. Mennucci, B. Polarizable continuum model, *WIREs Comput. Mol. Sci.* **2012**, *2*, 386–404.

S7. Daina, A.; Michielin, O.; Zoete, V. SwissADME: A free web tool to evaluate pharmacokinetics, drug-likeness and medicinal chemistry friendliness of small molecules. *Sci. Rep.* **2017**, *7*, 42717. DOI: 10.1038/srep42717

S8. Tetko, I.V.; Tanchuk, V.Y. Application of associative neural networks for prediction of lipophilicity in ALOGPS 2.1 program. *J. Chem. Inf. Comput. Sci.* **2002**, *42*, 1136–1145. (http://www.vcclab.org/web/alogps/, accessed December 2018)

S9. Frisch, M.J. Trucks, G.W., Schlegel, H.B., Scuseria, G.E., Robb, M.A., Cheeseman, J.R., Scalmani, G., Barone, V., Mennucci, B., Petersson, G.A., et al. *Gaussian 09, Revision D.01*; Gaussian, Inc.: Wallingford, CT, USA, 2009.

S10. Pamidighantama, S.; Nakandala, S.; Abeysinghe, E.; Wimalasena, C.; Rathnayakae, S.; Marru, S.; Pierce, M. Community science exemplars in SEAGrid Science Gateway: Apache Airavata based implementation of advanced infrastructure, *Procedia Comput. Sci.* **2016**, *80*, 1927–1939.

S11. Shen, N.; Fan, Y.; Pamidighantam, S. E-Science infrastructures for molecular modeling and parametrization, *J. Comput. Sci.* **2014**, *5*, 576–589.

S12. Dooley, R.; Milfeld, K.; Guiang, C.; Pamidighantam, S.; Allen, G. From proposal to production: Lessons learned developing the computational chemistry grid cyberinfrastructure, *J. Grid Comput.* **2006**, *4*, 195–208.

S13. Milfeld, K.; Guiang, C.; Pamidighantam, S.; Giuliani. J. Cluster computing through an application-oriented computational chemistry grid. Proceedings of the 2005 Linux Clusters: The HPC Revolution, April 2005 (accessed on 15 December 2018).

S14. This work used the Extreme Science and Engineering Discovery Environment (XSEDE), which is supported by National Science Foundation grant number OCI-1053575.

© 2019 by the authors. Submitted for possible open access publication under the terms and conditions of the Creative Commons Attribution (CC BY) license (http://creativecommons.org/licenses/by/4.0/).
